# Porcine Breast Extracellular Matrix Hydrogel for Spatial Tissue Culture

**DOI:** 10.3390/ijms19102912

**Published:** 2018-09-25

**Authors:** Girdhari Rijal, Jing Wang, Ilhan Yu, David R. Gang, Roland K. Chen, Weimin Li

**Affiliations:** 1 Department of Biomedical Sciences, Elson S. Floyd College of Medicine, Washington State University, Spokane, WA 99202, USA; girdhari.rijal@wsu.edu; 2 Tissue Imaging and Proteomics Laboratory, Washington State University, Pullman, WA 99164, USA; jing.wang6@wsu.edu (J.W.); gangd@wsu.edu (D.R.G.); 3 School of Mechanical and Materials Engineering, Washington State University, Pullman, WA 99164, USA; ilhan.yu@wsu.edu (I.Y.); roland.chen@wsu.edu (R.K.C.)

**Keywords:** porcine, breast cancer, extracellular matrix, hydrogel, tissue matrix scaffold, scaffold, 3D culture

## Abstract

Porcine mammary fatty tissues represent an abundant source of natural biomaterial for generation of breast-specific extracellular matrix (ECM). Here we report the extraction of total ECM proteins from pig breast fatty tissues, the fabrication of hydrogel and porous scaffolds from the extracted ECM proteins, the structural properties of the scaffolds (tissue matrix scaffold, TMS), and the applications of the hydrogel in human mammary epithelial cell spatial cultures for cell surface receptor expression, metabolomics characterization, acini formation, proliferation, migration between different scaffolding compartments, and in vivo tumor formation. This model system provides an additional option for studying human breast diseases such as breast cancer.

## 1. Introduction

Hydrogel is a gel formed by networks of polymer chains, which have the ability to retain the water and gelatinize easily through various cross-linking processes. It has a wide range of applications ranging from biomedical research, tissue engineering, and clinical utilization to drug delivery [1,2,3,4]. Hydrogels are commonly classified into two major categories, synthetic and natural, based on the origin and biochemical properties of the source materials used to produce the gel. Although synthetic hydrogels have been used in research fields for decades owing to many advantages, including long service life, high capacity of water absorption, high gel strength, easy availability, low cost, comparably simple fabrication process, adjustable signaling inputs by integration of different extracellular matrix (ECM) proteins or polypeptides, and experimental reproducibility [3], a growing trend of using hydrogels derived from natural, especially tissue-specific native biomaterials in biomedical and bioengineering applications has become increasingly robust recently [5].

Among native tissue-derived hydrogels, collagen and the laminin-rich ECM (lrECM or Matrigel) [6,7] have been extensively used in various bioassays and experiments for phenotypic and mechanistic studies of human diseases as well as for tissue engineering [8,9,10,11]. These studies have contributed substantially to our understanding of cell biology on substrata that resemble those existing in native environments, which are beyond the supporting capacity of plastic and synthetic polymeric surfaces. The compositional differences of the substrata that cells live on are important for cell adhesion, migration, and growth since different cell surface receptors are expressed in response to their interacting proteins or particles within the matrices for extracellular and intracellular signaling and other biological functions. To this end, collagen and the compositionally under-defined mouse sarcoma lrECM may induce cellular phenotypes different than those seen in cells grown on tissue-specific matrices since the levels and types of collagen and other components within ECM of different organs or tissues are quite distinct [12,13,14].

In an effort to promote tissue-specific scaffolding tools available to study breast cancers, we recently reported a tissue matrix scaffold (TMS) system generated using mouse mammary fat pad (MFP) ECM [15]. Both hydrogel and porous TMS were derived from the same ECM source and used in spatial breast tumor modeling and drug testing, representing an ideal platform for consistent studies from in vitro to in vivo without switching to different culturing matrices. In this current work, we present the biochemical composition of the ECM of female porcine breast fatty tissues and introduce the fabrication of hydrogel and porous scaffold using the purified ECM. Finally, select applications of the scaffolds in breast cancer research are exhibited.

## 2. Results and Discussion

### 2.1. Extraction and Identification of Porcine Breast Tissue ECM Proteins

**Figure 1:**
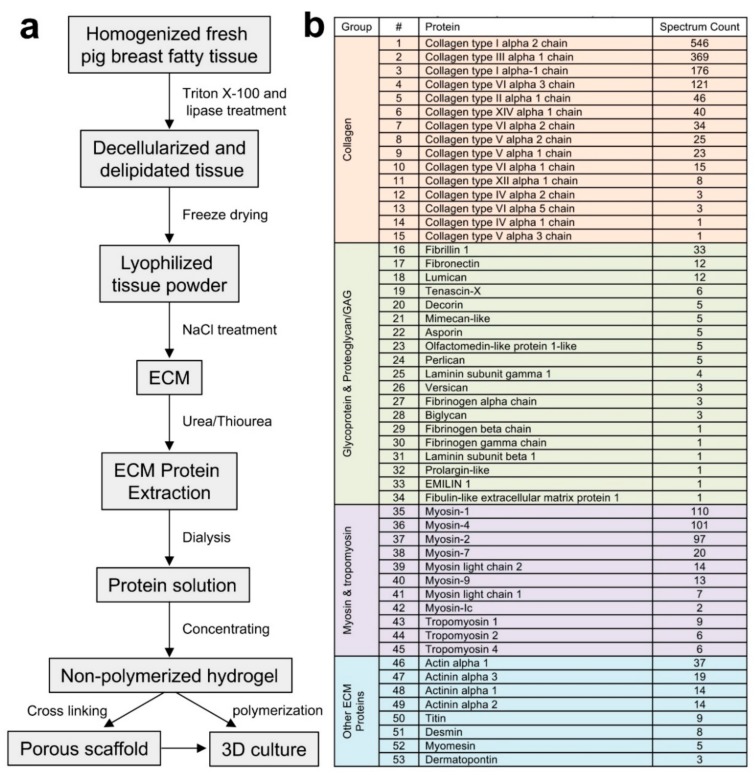
Extracellular matrix (ECM) protein extraction and identification from porcine breast fatty tissues. (**a**) Outline of the major steps of the breast ECM protein extraction and downstream applications. (**b**) The major ECM proteins of porcine breast fatty tissues.

Proper handling of fresh tissues is critical for minimal degradation and maximal identification of native proteins within the tissues. Homogenization of the fresh porcine breast fatty tissues directly obtained from local slaughter house was conducted in a precooled homogenizer, with the container holding the tissues embedded in ice and sliced tissues mixed with ice-cold water as described in the materials and methods. The homogenized tissues were then decellularized with the non-ionic detergent triton X-100 or the zwitterionic detergent CHAPSO to remove the cellular contents while preserving the native and active states of the ECM proteins. Since porcine breast tissues contain rich fat that is difficult to remove compared to that within the mammary tissues of mice and human, we applied lipase in the detergent solution to maximize lipid removal and the decellularization efficiency (Figure 1a). The detergents and lipase were removed with multiple rounds of washing with ddH_2_O. The resulting ECM dry weight was about 2% of the total fresh tissues used for the extraction. Whole ECM protein extraction from the ECM was carried out using a gradient of concentrations of urea and thiourea solutions to solubilize proteins that could be dissolved under different urea solution conditions due to their sizes and native conformational states. After dialysis and concentration of the protein extract, the total protein amount within the extract was measured at about 90% of the ECM used for the extraction. 

The proteomic composition of the porcine ECM was identified using liquid chromatography coupled with tandem mass spectrometry (LC-MS/MS). The majority of ECM proteins detected were different types of collagen, representing about 70% of the major proteins listed (Figure 1b), with collagen I and III being the most abundant (~77% based on single chain ratios) among the total collagen content. While the sum of glycoproteins and proteoglycans accounted for about 5% of the ECM major proteins, the content of myosin and tropomyosin was about 19%, with other ECM proteins comprising the remaining 6%. Interestingly, when we compared these data with the proteomic profiles of mouse normal MFP ECM that we reported previously [15], the ECM of rat mammary tissues [12] and the ECM of human breast tissues adjacent (considered to be normal) to tumors [16], it appeared that the ECM total collagen content of the human breast tissue ECM (about 80–85%) was in between of that of the porcine breast ECM and the rodent mammary ECM (about 85–90%). Another intriguing result was that higher myosin and tropomyosin content was detected in the porcine breast ECM compared to that of the rodent mammary ECM (less than 0.5%) and of the human breast ECM (about 1%) [16]. The amounts of the overall glycoproteins and proteoglycans within the ECM of porcine, rodent, and human were similar at about 5–7%. These ECM compositional differences of the porcine, mouse, and human breast tissues could be due to the anatomical and physiological natures of the native tissues that are related to the functions of the tissues in each specific species. It is noteworthy that the reproductive cycle seems to play a role in mammary tissue ECM compositional changes [12,17]. Additionally, the differences in the buffers and methods used to extract native ECM proteins clearly have an impact on the types and amounts of proteins identified [12,15,16,18]. Future proteomic analyses of mammary ECM compositions of the aforementioned and additional species using the same extraction and analytical methods will potentially identify novel discrepancies upon cross comparing the data sets obtained from the same experiments.

### 2.2. Generation and Characterization of the Porcine Breast ECM Hydrogel Scaffold

**Figure 2:**
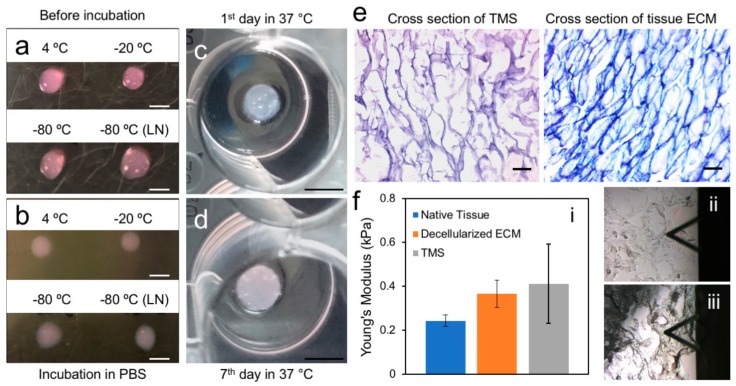
Physical and structurally properties of the porcine breast ECM hydrogel and porous TMS. (**a**) The ice-cold ECM hydrogel stored under different conditions (hydrogels kept in −20 °C and −80 °C were thawed in ice at 4 °C) dispensed on the clean and dry plate before polymerization tested and subjected for polymerization at 37 °C for 30 min. (**b**) The polymerized hydrogel was immersed in 1× PBS or DMEM and placed in 37 °C incubator (5% CO_2_) for stability test. Scale bars, 4 mm for (**a**,**b**). (**c**,**d**) The sustainability of the porous scaffolds generated from the ECM hydrogel was assessed by submersing the scaffolds in PBS or DMEM and incubation at 37 °C (5% CO_2_) for 7 days. Scale Bars, 6 mm. (**e**) H&E staining of the cross sections of the porous scaffolds and decellularized porcine breast fatty tissue ECM. Scale bars, 100 μm. (**f**) AFM measurement of the mechanical properties of porcine native breast tissues, decellularized breast ECM, and porous TMS: (**i**) Young’s moduli of the samples; (**ii**) and (**iii**) Illustrations of the positions of the probe tips on the decellularized ECM and TMS samples, respectively.

To assess the impact of storage temperature of the ECM hydrogel on polymerization, the gels (8 mg/mL) directly stored at 4, −20, −80 °C, and flash froze in liquid nitrogen followed by storing at −80 °C were thawed on ice and dropped onto the bottom of a 100 mm tissue culture dish, which was then placed in a 37 °C incubator for polymerization. After 30 min of incubation, the gels from the different stocking conditions all gelatinized (Figure 2a). When the polymerized gels were submersed in 1× PBS or DMEM, they retained their initial shapes and remained undissolved during the 10-day testing period (Figure 2b). To maximize the preservation of the ECM proteins in their native forms and avoid protein degradation, we used liquid nitrogen flash freezing followed by storing at −80 °C as a standard approach for long-term stocking of the hydrogel solutions at different concentrations. Short-term storage on ice or at 4 °C for up to four weeks did not seem to affect the polymerization of the hydrogel and its performance in experiments as described below.

Generation of porous TMS using the ECM hydrogel was achieved using a combination of a gas foaming method [19] and a freeze-gelation approach [20] with modifications as described in the methods. This technique not only induced gelation of the hydrogel but also allowed production of interconnecting porous structures within the solidified gel. The sustainability of the fabricated porous scaffolds under regular tissue culture conditions (37 °C, 5% CO_2_) was evaluated in 1× PBS or DMEM for 7 days. Our results showed that the porous scaffolds were stable and retained their shape during the period of testing (Figure 2c,d). Hematoxylin and eosin (H&E) staining of the cross sections of the scaffolds showed inter-connective pores at the sizes of about 100–200 μm (Figure 2e, left panel), highly resembling those of the decellularized porcine breast fatty tissue ECM (Figure 2e, right panel).

In order to evaluate the mechanical resemblance of the reconstituted scaffolds to that of the matrix of native tissues, we used AFM to measure native porcine breast tissues, decellularized porcine breast ECM, and porous scaffolds generated using porcine breast ECM hydrogel. The AFM testing method on the stiffness of biological tissue structures, cells, and specific regions of ECM is not standardized. Thus, previous reports have shown different results depending on their individual testing conditions [21,22]. On a large scale, measurements of decellularized organ matrices with AFM have been reported [23,24]. On a fine scale, mechanical properties of breast cancer cells and their structures have been investigated using AFM [25]. The probe tip size, indentation rate, and maximum force applied to samples should be optimized based on the mechanical strength of the samples. Thus, we optimized our testing parameters specifically for the breast tissues as described in the methods. The positioning of the probe tip for indention on the decellularized ECM and TMS has been illustrated in Figure 2f-ii,f-iii.

Our AFM results showed that the average Young’s modulus of the native porcine breast tissues was 0.243 ± 0.027 kPa (Figure 2f–i), which corresponded to a similar range of the compliance of human normal breast tissues [25,26] and is about 45–50% higher than that of mouse normal mammary tissues [27,28]. The Young’s modulus of the decellularized porcine breast ECM was 0.366 ± 0.061 kPa (Figure 2f–i), which is about 50% higher than that of the porcine breast native tissues and stiffer than decellularized mouse normal mammary fatty tissues [28]. A similar trend of higher Young’s modulus in decellularized tissues than in native tissues was observed in AFM measurement of human liver samples [29]. Porous TMS generated using ECM hydrogel at the concentration of 40 mg/mL (data for the concentrations of 20 mg/mL and 60 mg/mL were not shown) demonstrated an average Young’s modulus of 0.411 ± 0.180 kPa (Figure 2f–i), which is close to that of the decellularized porcine breast ECM. These data collectively indicate that the structural and mechanical properties of the reconstituted porous TMS highly resemble those of the decellularized native ECM and are suitable for spatial tissue cultures that closely mimic native breast tissue microenvironment.

### 2.3. ECM Support of Cell Surface Receptor Expression and Metabolomes in Spatial Culture

**Figure 3:**
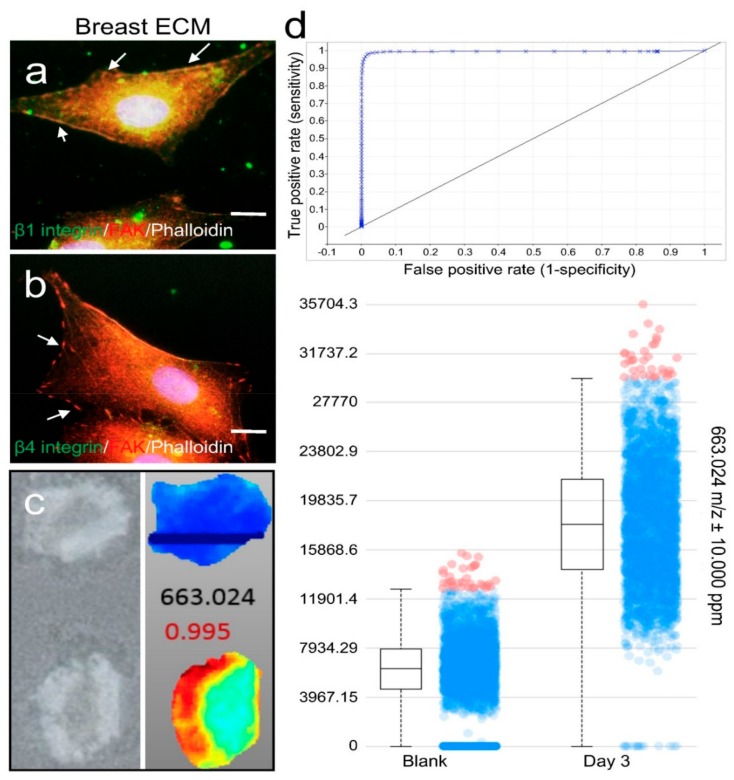
Membrane surface receptor expression and metabolomic analysis of cells grown on porcine breast fatty tissue ECM hydrogel. Integrin β1 (**a**) or β4 (**b**) receptor (green) and FAK (red) expression in MM231 cells cultured on the ECM hydrogel-coated coverslips were assessed by IF. Phalloidin staining (white) of F-actin was used to contour the cells. Scale bars, 10 μm. (**c**) MS imaging of cross sections of the medium-conditioned blank (upper panels) and MM231 cell culture-laden (bottom panels) hydrogel scaffolds. (**d**) Examples of ROC (upper) and box-and-whiskers (bottom) plots from the MS imaging analysis, showing data for a compound with *m*/*z* 663.024, where differences between the hydrogel samples cultured with or without MM231 cells are clearly evident.

Our proteomics data showed that collagen I and III were the major protein components, whereas the main basement membrane protein laminin only accounted for a minimal amount of the porcine breast tissue ECM. Therefore, we carried out immunofluorescence (IF) staining of integrin β1 and β4 plasma membrane receptors for collagen I/III and laminin, respectively, in MM231 cells grown on the breast ECM hydrogel-coated glass coverslips for 24 h. The expression of focal adhesion kinase (FAK) on the surface of the cells was also immunostained to serve as an indicator of the adhesion sites of the cells. Our confocal fluorescence microscopy results showed that high levels of integrin β1 and low levels of integrin β4 were observed in the cells cultured on the hydrogel matrix (Figure 3a,b), indicating that the cells attached to the matrix through integrin β1 and collagen I/III interactions.

To assess the capability of the porcine breast ECM hydrogel in capturing the metabolites secreted from cells grown on it, 4% porcine breast ECM hydrogel was added into the wells of 96-well plates at a thickness of 4 mm, followed by inserting porous TMS illustrated in Figure 2c (2-mm thick, 6-mm diameter, 100-μm pore size) into the surface section of the gel before its polymerization in a 37 °C incubator. A total of 1.0 × 10^4^ MM231 cells per well were seeded on the top of the porous scaffold and cultured in RPMI 1640 medium (Corning; 1×; 2 g/L of d-glucose; 10% FBS) under optimal conditions (37 °C, 5% CO_2_) for 3 days. The hydrogel samples underneath the porous scaffolds were collected, cross sectioned (Figure 3c, left panel) and processed for mass spectrometry (MS) analysis of the metabolites collected within the gels as described in the methods. 

Based on our MS spectral data, metabolite distribution patterns and relative abundance levels were determined using the SCiLS Lab MS imaging analysis software, which also grouped compounds (detected as ions with specific *m*/*z* values) into distinct ion distribution patterns across the scaffolds for comparison. A total of 41 distinct chemical features (i.e., the mass over charge ratios detected in MS) were selected based on their abundance distribution profiles and the positive scores (close to 1) resulted from the receiver operating characteristic (ROC) curve analyses in the MM231 cell culture-laden hydrogel samples compared to the medium-containing blank hydrogel samples. As illustrated by one of the forty-one results, the compound with *m*/*z* 663.024 was absent from the blank scaffold (Figure 3c, right panel, top) but showed high accumulation with a distinct pattern of being more abundant near the edges of the ECM hydrogel scaffold than in the center. This compound had a ROC value of close to 1 (highly positive; Figure 3d, top panel). The box plot for this compound (Figure 3d, bottom panel) further demonstrated its preferential accumulation within the hydrogel samples of the MM231 cell cultures. Similar results were found in all 41 ions (data not shown), i.e., higher specific chemical abundances were detected in the hydrogel samples of the MM231 cell cultures with high ROC scores. Four box plots for the ions with top ROC scores from the 41 individual groups were shown in Figure S1, which had *m*/*z* ratios at 663.024, 531.060, 839.043, and 768.371 with the ROC scores of 0.995, 0.996, 0.994 and 0.988, respectively. Together, these data demonstrate that the porcine breast ECM hydrogel scaffolds support matrix-associated cell membrane receptor expression and secretion of metabolites from the cells grown in the tissue-mimicking 3D space.

### 2.4. Applying Porcine Breast ECM Scaffold in Support of Spatial Cell Proliferation, Coculture of Cancer Cells and Stromal Cells, and Tumor Formation

**Figure 4:**
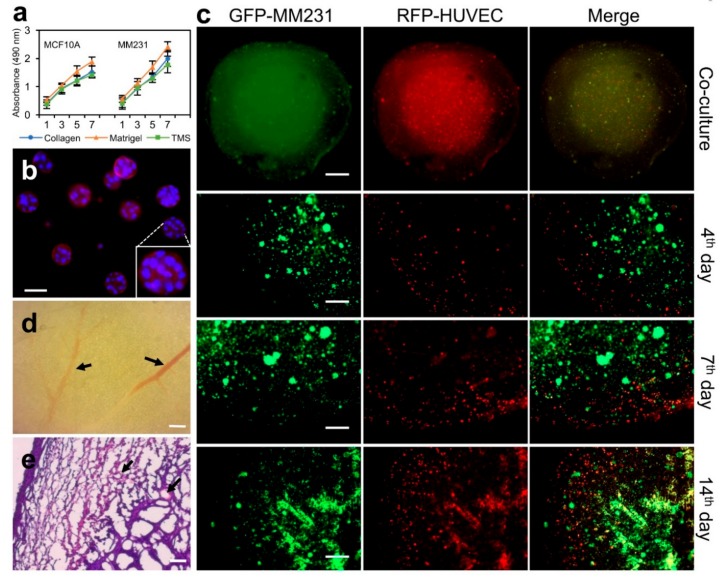
Applications of porcine breast ECM hydrogel and porous scaffolds in proliferation, migration, and tumor formation experiments. (**a**) Proliferation of MCF10A or MM231 cells on the porcine breast ECM hydrogel. Error bars, mean ± SD. (**b**) MCF10A cell acini formation within the ECM hydrogel. (**c**) Compartmental culture of MM231 cells and HUVECs to assess cross-compartmental migration of different types of cells. (**d**) The vasculature of a whole mount cross section of breast tumors from mice MFP implanted with MM231 cell-laden porous TMS derived from the porcine ECM hydrogel. (**e**) H&E staining of the cross sections of mice breast tumors developed from the cancer cell-laden TMS. Black arrows in d and e indicate blood vessels. Scale bars, 50 μm for (**b**); 1 mm—upper first panel (**c**) & 500 µm—lower three panels (**c**); 200 μm for (**d**,**e**).

To evaluate the proliferation of mammary epithelial cells on porcine breast ECM hydrogel, normal MCF10A or MM231 cells were seeded in 96-well plates coated with or without the porcine breast ECM hydrogel, collagen, and Matrigel, and cultured under optimal conditions (37 °C; 5% CO_2_) for 7 days. Cell proliferation was measured using WST-1 reagent (Sigma-Aldrich, St. Louis, MO, USA) at different time points (Day 1, 3, 5, 7). Our results showed that both type of the cells proliferated faster on Matrigel compared to those on collagen and ECM hydrogel (Figure 4a), possibly due to the compositional nature of Matrigel, which contains growth factors and underdefined cellular components derived from Engelbreth–Holm–Swarm (*EHS*) *sarcoma* sources [15,30]. In contrast, the growth of the cells cultured on the porcine breast ECM hydrogel is even slower than those on collagen but at comparable levels, suggesting certain degree of similarities of the two types of gels on supporting cell population expansion.

The spatial growth and proliferation of mammary epithelial cells within the breast ECM hydrogel were further assessed using an acini formation assay. Briefly, 1 × 10^4^ MCF10A cells were mixed into 200 μL of 2% porcine breast ECM hydrogel and cultured on 8-well chamber slide under optimal conditions as described above for 7 days. Acini formation was assessed using light microscopy, and the structures of the acini were characterized with phalloidin and DAPI IF staining followed by confocal microscopy. Our results showed that MCF10A cells formed acini at different sizes, with larger ones having hollow centers (Figure 4b). Compared to the traditional acini formation assay using Matrigel, the current method applies fewer matrix materials, simplified procedures, and shorter culture times. Importantly, the purified porcine total ECM hydrogel contains neither growth factors nor tumor cell products and has well-defined ECM proteins, lending promise for low background tissue cultures and adjustable options for adding desired culturing components within culture medium.

In order to observe the spatial expansion of cancer cell population and recruitment of stromal cells, we have devised a coculture system using the porcine breast ECM hydrogel and porous TMS (Figure 2c,d) derived from the hydrogel. 1 × 10^4^ GFP-MM231 cells were seeded on the porous scaffold (round, 2 mm thick, 4 mm diameter) placed in a well of 96-well plates. A layer of the ECM hydrogel (8 mg/mL) containing 1 × 10^4^ RFP-HUVECs (human umbilical vein endothelial cells) was covered on top of the porous scaffold (Figure 4c, top panels). After polymerization of the gel, 100 μL of 1× DMEM containing 10% FBS was added into the well (replicate samples were prepared). The two types of cells within the different yet mutually accessible compartments were cultured for 14 days. The distribution and migration of the cells within the co-culture system were imaged over time using confocal microscopy. Our data showed that both MM231 cells and HUVECs progressively increased their numbers and migrated out of their initial living compartment into adjacent areas (Figure 4, middle and bottom panels). The interaction of the two types of cells was also increased, as exhibited by the yellow overlapping regions. Clearly, this compartmental culture approach using hydrogel and porous scaffold derived from the same native tissue ECM allows for the observation of certain cellular phenotypes, such as spatial cell migration and interaction, in an advanced tissue-mimicking environment that could be difficult to be captured in live tissues or other nontissue-specific culture models.

We next tested the efficiency of the porcine breast ECM scaffold in supporting tumor formation in vivo. 1 × 10^5^ MM231 cells were seeded on a spherical porous TMS (4 mm diameter) and cultured for 24 h under optimal conditions as described before. Triplicate samples were prepared for the experiment. Then, the scaffolds were implanted separately into the mammary fat pad (MFP, 4th nipple region from the rostral side) of eight-week-old nulliparous NOD/SCID female mice. Tumor development was observed over a period of 4 weeks. The sizes of the tumors were dynamically measured using a caliper and reached an average of about 2-cm diameter at the end of week 4 post-implantation, at which point the tumors were collected, formalin fixed, cross-sectioned (10 μm thick), and H&E stained for histological examination as we reported previously [15]. Our histological data showed that visible blood vessels had been grown into the tumors in most of the regions of the outer half of the tumor bodies (Figure 4d,e, arrows), which were filled with mixtures of cancer cells and stromal cells clustered within or overlapped with ECM structures (Figure 4e). This data indicates that the porcine breast ECM-based scaffold represents another tissue-specific platform, in addition to mice ECM-based scaffolds [15], to support consistent formation of breast tumors in animals.

## 3. Materials and Methods

### 3.1. Tissue Collection and Decellularization

The fresh mammary tissues from female pigs were collected aseptically from a local slaughter house, where the research purpose of using the designated tissues was informed. The animal use protocol (#04965) has been approved (01/30/2017) by WSU Institutional Animal Care and Use Committee (IACUC). The tissues on ice were immediately transferred to the lab, sliced into small pieces, and homogenized in sterilized ice-cold deionized distilled water (ddH_2_O). After centrifugation (10,000 RPM/17,000 RCF; 30 min) at 42 °C (Pig fat is relatively sticky and melts only above 37 °C) of the homogenized tissues, the supernatant together with the fat were discarded. The centrifugation process was repeated for a couple of rounds to maximally remove the fat until there was visible oil droplets on the surface of the supernatant. The homogenized tissues were then mixed with a sufficient amount (at least 10 times more than the homogenized tissue volume) of 0.1% Triton X-100 or 4% CHAPSO with constant agitation at room temperature for 12 h and centrifuged as described above. This decellularization process was repeated one more time. The pelleted ECM was transferred into 50 mL conical tubes containing 0.1% Triton X-100, cOmplete mini protease inhibitor cocktail (Sigma-Aldrich, St. Louis, MO, USA), and lipase (1 mg/g of ECM), and incubated at 37 °C with constant rotation for 12 h, followed by several rounds of washing with ddH_2_O and centrifugation to ensure complete removal of Triton X-100, protease inhibitors, and lipase from the samples. The ECM was lyophilized and assessed for decellularization efficiency using the removal of DNA content (<50 ng per milligram of dried ECM) and the maximal retention of collagen and glycosaminoglycan (GAG) within the ECM as parameter references as described previously [15].

### 3.2. Extraction of Porcine Breast Tissue ECM Proteins

The lyophilized ECM was pulverized by grinding in liquid nitrogen, followed by treating the ECM powder twice with 3.4 M NaCl buffer (99.25 g NaCl, 6.25 mL of 2 M Tris Base, 0.75 g EDTA, and distilled water to 500 mL, final pH 7.4) at 4 °C for 15 min. The ECM was pelleted by centrifugation and homogenized in 2 M urea buffer (60 g Urea, 3.025 g Tris Base, 4.5 g NaCl, distilled water to 500 mL, pH 7.4) at 4 °C overnight. The sample was then centrifuged at 13,000 RPM/28,720 RCF for 30 min. The supernatant was collected and kept on ice. Further homogenization of the remaining ECM was followed using 4 M and 6 M urea buffer, respectively, and the supernatant from each extraction was collected and stored as described above. The remaining insoluble ECM was treated with increasing concentration of urea/thiourea (6 M/0.5 M; 6 M/2 M; 7 M/0.5 M; and 7 M/2 M) for 12 h at 4 °C, and supernatant collected as before. The insoluble ECM sediment was further homogenized with 8 M urea, and then with 2% of n-octyl β-d-glucopiranoside (OG) overnight at 4 °C. Again, the supernatant was collected after centrifugation at 13,000 RPM/28,720 RCF for 30 min. The urea concentrations of the different batches of the supernatants were brought to 2 M. Then the tissue ECM protein extracts were pooled together and dialyzed in cold TBS (6.05 g Tris Base, 9.0 g NaCl, total volume of 1 L, pH 7.4, with 5 mL of chloroform) for at least 2 h. Dialysis was repeated twice in cold TBS without chloroform for 12 h. Further dialysis with serum free 1× DMEM medium is optional. The sterile ECM protein solution was then concentrated using polyethylene glycol (PEG) and stored at −80 °C for future use.

### 3.3. Identification of the ECM Proteins and Data Analysis

Identification of the extracted ECM proteins was conducted as reported previously [15] . Briefly, the ECM proteins were solubilized in 8 M urea solutions containing 1% ProteaseMAX (Promega), subjected to Dithiothreitol (DTT) reduction, iodoacetamide (IAA) alkylation, trypsin digestion, followed by LC-MS/MS. The raw data was converted to mgf files, which were used to search against *Sus Scrofa* amino acid sequence database with decoy reverse entries and a list of common contaminants (81,280 total entries with 40,602 pig proteins from UniProt database downloaded 11_28_2017) using in-house Mascot search engine 2.2.07 (Matrix Science, Boston, MA, USA) with variable methionine and proline oxidation, and with asparagine and glutamine deamidation. Peptide mass tolerance was set at 15 ppm and fragment mass at 0.6 Da. Protein annotations, significance of identification and spectral based quantification was performed with the help of Scaffold software (version 4.4.1, Proteome Software Inc., Portland, OR, USA). Protein identifications were accepted if they could be established at greater than 80.0% probability within 1% false discovery rate and contained at least two identified peptides. Protein probabilities were assigned by the ProteinProphet algorithm [31].

### 3.4. Generation of Porous Scaffold Using the ECM Hydrogel

The porcine breast ECM hydrogel at the concentration of 40 mg/mL was mixed with sodium bicarbonate (final concentration 1 mg/mL), filled in desired culture vessels (for example 96-well plates), flash-frozen in liquid nitrogen, and stored in −80 °C overnight. The frozen hydrogel blocks were then immersed into precooled (−80 °C) ethanol bath containing 0.1 M acetic acid until there was no bubbles arising from the solidified gel (the processing time depends on the size of the frozen gel). The ethanol was replenished, and the scaffolds remained immersed for at least 24 h. Before cell culture, the scaffolds were washed three times with 1× PBS or tissue culture medium.

### 3.5. Atomic Force Microscopy (AFM)

AFM (Dimension Icon ScanAsyst, Santa Barbara, CA, USA) was used to determine the Young’s moduli of the samples. Three different types of samples were prepared for this study: porcine breast native fatty tissues, decellularized ECM of porcine breast native fatty tissues, and porous TMSs generated using hydrogel extracted from the decellularized ECM of porcine breast native fatty tissues as described above. Samples were embedded in optimal cutting temperature (OCT) compound and frozen in ethanol-dry ice bath, cross sectioned into 20 μm of thickness, mounted on silane-coated slides, and rinsed with 1× PBS and ddH_2_O to remove OCT. The samples were kept at 4 °C until AFM measurement. Before AFM, the samples were allowed to warm up to room temperature in a sealed plastic bag to prevent drying. AFM measurement of the mechanical properties of the samples was conducted through borosilicate spherical-shaped tips (Novascan, Boone, IA, USA) with a size of 5 μm and 0.06 N/m spring constant [25]. The spring constant of cantilever was matched with the stiffness of the porcine breast tissues or decellularized ECM samples to be tested. During AFM measurement, the maximum force was set to 2 nN. The total indentation depth was 1 µm with an indentation rate of 20 μm/s. For the native tissue samples, data points were collected by every 10 µm along the *x*-axis. For decellularized ECM and TMS, due to the porous structures of the samples, the data points were collected by manually selecting areas of interest to avoid probing inside pores. At least 7 different points were measured for each sample. The data was processed using Hertz’s model with Poisson’s ratio of 0.5 to determine the Young’s moduli of the samples. 

### 3.6. Cells and Tissue Cultures

Human breast cancer epithelial cells MDA-MB-231 (MM231) and normal breast epithelial cells MCF10A were purchased from ATCC, and GFP-MM231 cells and RFP-HUVECs were from Cell Biolabs and ANGIO-PROTEOMIE, respectively. The cells were cultured in optimal medium under 37 °C and 5% CO_2_ conditions as described previously [15,32].

### 3.7. Immunofluorescence Staining (IF)

IF staining of the cells cultured on coverslips coated with porcine breast tissue ECM hydrogel and confocal microscopy were carried out as described previously [33]. Integrin β1 (#MAB17782), integrin β4 (MAB4060), and FAK (#NBP1-84750) antibodies were purchased from Novus Biologicals (Littleton, CO, USA). Alexa Fluor 663 Phalloidin probe was purchased from ThermoFisher Scientific (#A22284) (Waltham, MA, USA).

### 3.8. Metabolome Analysis

The hydrogel samples collected from the tissue cultures were flash frozen, cross sectioned (10-μm thick) and mounted onto the cold indium tin oxide (ITO)-coated microscope slides (Figure 3c, left panel). Three experimental replicates were prepared, and three consecutive sections from an individual replicate were collected. A matrix solution of 2,5-dihydroxybenzoic acid (DHBA, 20 mg/mL) dissolved in water and methanol mixture (50:50 in volume) was applied to the tissue sections on the different slides using a TM-Sprayer. The DHBA-coated slides were analyzed using a Bruker SolariX 9.4T MALDI FTICR mass spectrometer (Bruker Daltonics, Billerica, MA, USA) to scan ions within a mass range of 150–3000 Da. Imaging acquisition was carried out using FlexImaging (Bruker Daltonics). The spectral analyses were performed using DataAnalysis (Bruker Daltonics) and SciLS Lab (SciLS, Bremen, Germany).

### 3.9. Cell Proliferation Assay

The growth and proliferation of the cells on TMS scaffolds were measured using WST-1 reagent as described previously [19,32]. Briefly, 2% of porcine breast ECM hydrogel, 100 µL was dispensed into each well of a 96-well culture plate and incubated at 37 °C for cross linking as described above, forming TMS scaffolds. MCF10A or MDA-MB-231 suspended in the respective culture media were seeded on the scaffolds (1 × 10^4^ per scaffold) and allowed to attach for 45 min as we described in our previous paper [19]. 100 µL of respective culture medium was then added and cultured under the optimal conditions (37 °C, 5% CO_2_), replacing media in every alternate day. The proliferation of the cells grown on the scaffolds was measured using WST-1 (Sigma-Aldrich) at the points indicated (1st, 3rd, 5th and 7th day). WST-1 solution (10 µL) was added at a 1:10 dilution into the cultures and incubated for 2 h. The supernatants of the cultures were collected and the colorimetric reactions that reflect the proliferation status were measured using a Synergy 2 microplate reader (BioTek, Winooski, VT, USA) for the absorbance at 490 nm. Error bars represent standard deviations (SD) of the means of three independent experiments. 

### 3.10. Data Availability

The MS proteomics and metabolomics data have been deposited to the ProteomeXchange Consortium via the PRIDE partner repository with the data set identifier PXD011011 (proteomics) and PXD010960 (metabolomics), respectively.

## 4. Conclusions

Porcine mammary ECM contains abundant collagen as well as other structural or compositional proteins similar to those in mouse [15] and human breast ECM [16]. Additionally, the physical structures of the decellularized mammary ECM of these different native tissue materials are also close to each other. These physicochemical resemblances of the native ECM across the different mammalian species potentially benefit our research involving the applications of the ECM in phenotypic, mechanistic, or pharmacological response studies of human diseases relevant to the ECM source organs or tissues. The tissue-specific native ECM-based studies will provide valuable and clinically relevant insights into the development of human cancers and other diseases. We expect to see a steady growth of using tissue-specific biomatrices to address disease-specific questions in the future.

## Supplementary Materials

Supplementary materials are available online at http://www.mdpi.com/1422-0067/19/10/2912/s1.
