# Supplementary material for: Porcine Breast Extracellular Matrix Hydrogel for Spatial Tissue Culture
Source: Int J Mol Sci. 2018 Sep 25;19(10):2912. doi: 10.3390/ijms19102912 (PMC6213433; doi:10.3390/ijms19102912)
Supplement: Supplementary file 1 [file ijms-19-02912-s001.pdf]

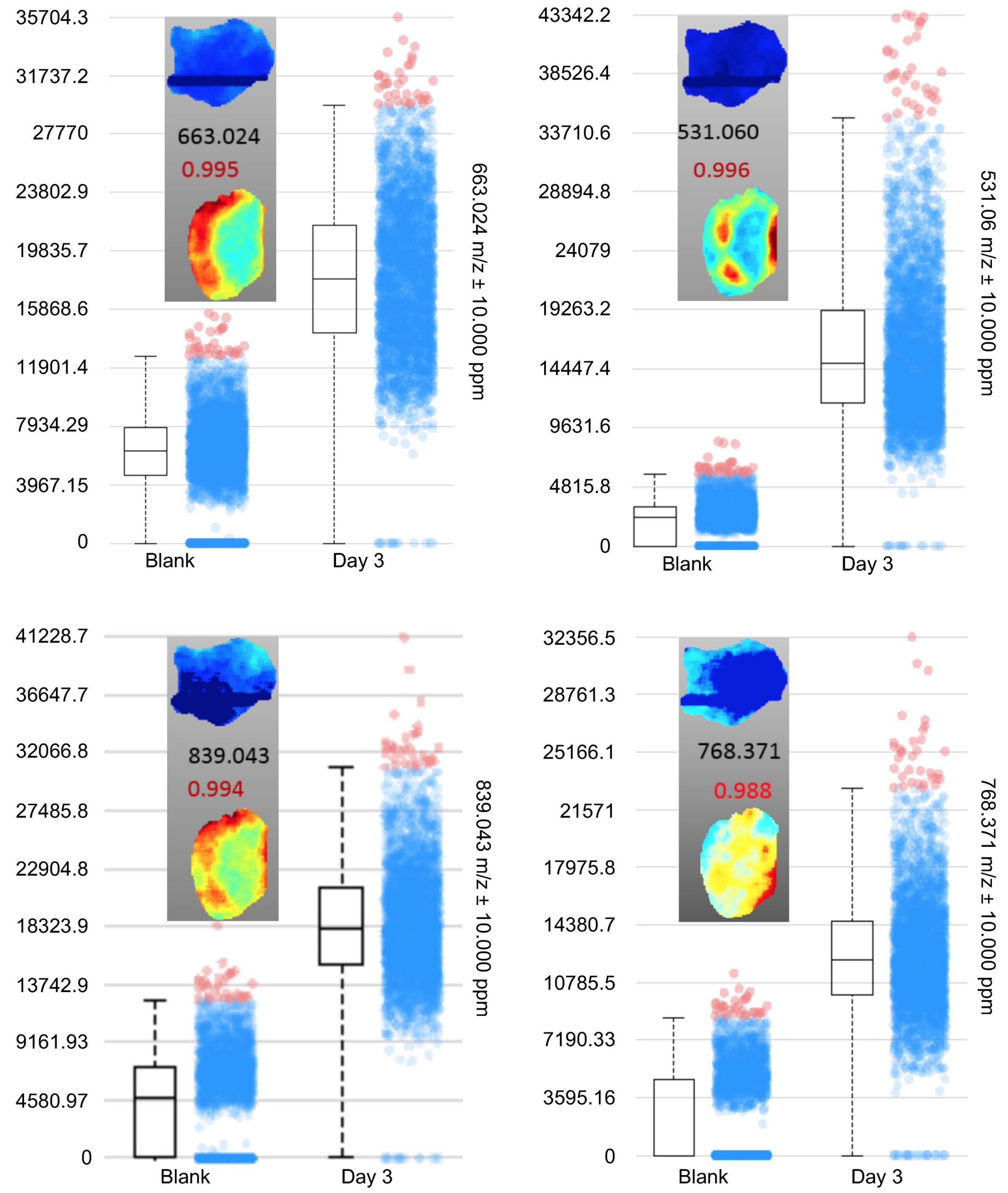

Figure S1: Box plots for the metabolomic ions with top ROC scores identified within the porcine breast ECM hydrogel samples of the MM231 cell spatial cultures. The four data panels were selected from 41 ion pairs based on the calculated threshold (~0.05) from SCiLS Lab analysis.
